# Cancer‐ and Chemotherapy‐Induced Musculoskeletal Degradation

**DOI:** 10.1002/jbm4.10187

**Published:** 2019-02-25

**Authors:** Kathleen M Sturgeon, Katlynn M Mathis, Connie J Rogers, Kathryn H Schmitz, David L Waning

**Affiliations:** ^1^ Department of Public Health Science Penn State College of Medicine Hershey PA USA; ^2^ Penn State Cancer Institute Hershey PA USA; ^3^ Department of Nutritional Sciences Penn State College of Health and Human Development University Park PA USA; ^4^ Department of Physical Medicine and Rehabilitation Penn State College of Medicine Hershey PA USA; ^5^ Department of Cellular and Molecular Physiology Penn State College of Medicine Hershey PA USA

**Keywords:** TUMOR‐INDUCED BONE DISEASE, BONE‐MUSCLE INTERACTIONS, CANCER, CHEMOTHERAPY

## Abstract

Mobility in advanced cancer patients is a major health care concern and is often lost in advanced metastatic cancers. Erosion of mobility is a major component in determining quality of life but also starts a process of loss of muscle and bone mass that further devastates patients. In addition, treatment options become limited in these advanced cancer patients. Loss of bone and muscle occurs concomitantly. Advanced cancers that are metastatic to bone often lead to bone loss (osteolytic lesions) but may also lead to abnormal deposition of new bone (osteoblastic lesions). However, in both cases there is a disruption to normal bone remodeling and radiologic evidence of bone loss. Many antitumor therapies can also lead to loss of bone in cancer survivors. Bone loss releases cytokines (TGFβ) stored in the mineralized matrix that can act on skeletal muscle and lead to weakness. Likewise, loss of skeletal muscle mass leads to reduced bone mass and quality via mechanical and endocrine signals. Collectively these interactions are termed bone‐muscle cross‐talk, which has garnered much attention recently as a prime target for musculoskeletal health. Pharmacological approaches as well as nutrition and exercise can improve muscle and bone but have fallen short in the context of advanced cancers and cachexia. This review highlights our current knowledge of these interventions and discusses the difficulties in treating severe musculoskeletal deficits with the emphasis on improving not only bone mass and muscle size but also functional outcomes. © 2019 The Authors. *JBMR Plus* published by Wiley Periodicals, Inc. on behalf of American Society for Bone and Mineral Research.

## Introduction

Bone loss and muscle weakness are significant sequelae of cancers metastatic to bone and of cancer therapy. Specifically, cancers of the breast, prostate, and lung have a high propensity for metastasis to bone, with 73%, 68%, and 36% of patients with advanced cancer developing a bony lesion, respectively.^1^ Estrogen‐receptor positive status has been identified as a potential risk for developing breast cancer bone metastases;^2^ however, a recent systematic analysis concluded that the primary risk factors for developing bone metastases in women with breast cancer are younger age, greater stage, and larger tumor size at diagnosis, whereas estrogen‐receptor status had no effect on bone metastasis risk.^3^ For patients diagnosed with prostate cancer, PSA levels ≥20 ng/mL, a Gleason score ≥8, and locally advanced disease are risk factors for developing bone metastases.^4^ In lung cancer, bone metastases are more commonly found in the adenocarcinoma subtype, whereas they are least common in small cell lung cancer.^5^ Whether the bone lesions are osteolytic (bone loss) or osteoblastic (bone formation) by X‐ray imaging, there is evidence of excess bone resorption in the majority of cancers metastatic to bone and increased risk of fractures that require surgery and spinal cord compression complications.^6^, ^7^ Cancer patients are also at increased risk of developing osteoporosis due to cancer treatment, so‐called cancer treatment‐induced bone loss (CTIBL).^8^, ^9^

Muscle weakness in patients with advanced cancer is associated with poor outcomes and exists as a spectrum that ranges from weakness in the absence of weight loss to profound muscle wasting and cachexia.^10^ Muscle weakness and loss of muscle mass affects between 15% and 80% of patients with cancer, depending upon tumor type and stage,^11^, ^12^ with the highest prevalence in those with advanced stages of cancer.^13^ Although the prevalence of combined muscle and bone loss in patients with cancer is unknown, it is logical to assume that they occur together relatively frequently given the importance of muscle‐bone cross‐talk in maintaining both tissue types.^10^

Bone loss and muscle weakness in cancer patients increase the risk of falls and fractures.^9^ In fact, a fivefold increase in fractures per year has been shown for women with newly diagnosed breast cancer receiving chemotherapy.^14^ These musculoskeletal events further negatively impact performance status, survival, and quality of life. Performance assessments of muscle function in cancer patients who received chemotherapy show slower chair‐rise time, reduced hand‐grip strength, and a decline in 12‐minute walk distance compared with healthy control individuals.^15^ Moreover, individual physician‐documented case reports show that lower‐extremity muscle weakness is a common complaint in patients receiving chemotherapy.^16^

The reduction in bone quality and muscle function are further exacerbated by inactivity often associated with these patients, which sets up a vicious cycle of increased immobility and reduced bone and muscle quality. In many cases, this reduces cancer treatment options, further eroding survival. Compounding the acute clinical impact of cancer metastases to bone and chemotherapy toxicities is the fact that these often cause chronic muscle weakness and exercise intolerance that can persist from months to years after remission of cancer.^15^, ^17^

## Bone Loss in Cancer Patients

Long‐term sequelae of cancer therapy include an increased risk for developing osteoporosis. Several anticancer therapies (hormonal and nonhormonal) have the potential to promote bone loss through direct dysregulation of bone turnover and indirect mechanisms such as hypogonadism and nephrotoxicity.^18^ Such therapies include endocrine therapies for breast cancer, which mitigate the effects of estrogen; androgen deprivation therapy (ADT) for prostate cancer; and antineoplastic drugs such as platinum‐derived compounds (cisplatin), alkylating agents (ifosfamide, cyclophosphamide, doxorubicin), antimetabolites (methotrexate), glucocorticoids, and targeted therapies. Additionally, other interventions for cancer patients such as radiation therapy, gonadal ablation, bilateral orchiectomy, and oophorectomy also result in bone loss.^9^ Ultimately, increased bone resorption and turnover can lead to osteopenia, osteoporosis, and resultant increases in fracture risk and mortality.^18^

Much current work is focused on understanding cancer therapy‐induced bone loss and the best approaches to preventing or reducing bone loss. In particular, preclinical studies of breast cancer bone metastases have shed much light on this topic. Doxorubicin and carboplatin chemotherapies have been used to study musculoskeletal changes and have revealed that these agents alone cause significant reduction in bone volume.^19^, ^20^ The combination therapy Folfiri (5‐fluorouracil, leucovorin, and irinotecan) also causes reduced bone volume.^21^

Increased risk of fracture arises from low bone mass, low bone strength, microarchitectural disruption, and increased skeletal fragility. Further, fragility fractures (fractures that occur without trauma) are commonly found in the spine (vertebral compression fractures), hip, and wrist. The World Health Organization (WHO) has defined osteopenia and osteoporosis based on dual‐energy X‐ray absorptiometry (DXA) measurements,^22^ and individuals with low bone mass are at increased risk for fracture. Although age‐related bone loss and increased fracture risk is a significant issue in the general population, it is even more concerning for cancer patients and survivors. Due to cancer therapy, cancer patients and survivors suffer from accelerated bone loss. Indeed, rates of bone loss from cancer therapy can be 10 times higher than in the general population.^23^, ^24^, ^25^, ^26^

As of 2016, there were more than 7.3 million male cancer survivors and 8.1 female cancer survivors.^27^ Prostate cancer survivors comprise 44.8% of male cancer survivors, and breast cancer survivors comprise 43.6% of female cancer survivors. Survivors with these cancer types are at the most significant risk of developing bone metastases, and further, these cancer types are often treated with therapies that negatively impact bone mass.^28^, ^29^

### Breast cancer

The majority of breast cancer patients have pathology consistent with overexpression of hormone receptors (ER+/PR+).^30^ Thus, the majority of breast cancer survivors are treated with endocrine therapies. Aromatase inhibitors (AIs) block estrogen production. The most common AIs are anastrozole, letrozole, and exemestane. Other drugs block estrogen's effects by binding to the estrogen receptor. Tamoxifen is a selective estrogen receptor modulator (SERM), and fulvestrant is a selective estrogen receptor degrader (SERD). Surgical interventions such as ovarian ablation will eliminate ovarian function and estrogen production. Lastly, ovarian suppression can also be induced through drugs such as goserelin or leuprolide.

Premenopausal breast cancer patients receiving ovarian suppression in the form of goserelin have increased bone loss (10.5%) 6 years after a 2‐year treatment regimen compared with women receiving a traditional adjuvant chemotherapy regimen of cyclophosphamide, methotrexate, and 5‐fluorouracil (6.5%). The onset of bone loss from premature menopause is sudden (6 months of treatment)^31^ and significant (21% decreased bone density compared with age‐matched eumenorrheic women).^32^, ^33^ In postmenopausal women, several large studies have been completed examining risk of bone fracture in breast cancer patients who received tamoxifen, AIs, or no endocrine treatment. A recent meta‐analysis examined 21 independent studies in women aged 65 and younger, with stage 1 to 3 breast cancer, treated with tamoxifen or AIs, and showed: 1) fracture risk was not elevated by tamoxifen use; 2) AIs increased fracture risk by 17% compared with women who did not receive AIs; and 3) AIs increased fracture risk by 35% compared with women on tamoxifen.^34^ It was also observed that women on AIs have higher fracture risk during treatment than after treatment end.^34^ Indeed, long‐term survivors of early stage breast cancer (postmenopausal at diagnosis) are not at greater risk of osteoporotic fractures compared with age‐matched women without breast cancer.^35^

### Prostate cancer

Significant bone loss can occur in men with prostate cancer who are treated with ADT due to hypogonadism.^36^ It is important to note that guidelines from the American Urological Association recommend that primary ADT alone not be included among standard options for the initial treatment of men with localized prostate cancer.^37^, ^38^ Radiation therapy is usually combined with ADT for improved outcomes in men with intermediate or high‐risk prostate cancer.^39^, ^40^, ^41^ Further, it is unclear if adding chemotherapy in combination with radiation therapy and ADT will improve outcomes in the adjuvant setting.^42^, ^43^ In the metastatic setting for prostate cancer, ADT is the main therapeutic approach with combination of antiandrogens and chemotherapy determined by clinical presentation. Other treatments that lower androgen levels include orchiectomy (surgical castration); luteinizing hormone‐releasing hormone agonists (leuprolide, goserelin, triptorelin, and histrelin); an inhibitor of the CYP17 enzyme, abiraterone; and antiandrogens (flutamide, bicalutamide, nilutamide, enzalutamide, and apalutamide).

Loss of bone mineral density can be detected after 6 to 9 months of ADT, and longer therapy confers a higher risk.^44^, ^45^, ^46^ Annual declines of bone mineral density are 2% to 8%.^47^, ^48^ Osteoporotic skeletal fractures occur in up to 20% of men within 5 years of starting ADT.^49^ Although several small studies have shown biclutamide, a nonsteroidal antiandrogen, to have more favorable bone health outcomes,^50^, ^51^ the prevailing consensus is that hypogonadism induced through treatment of prostate cancer is a contributor to reduced bone mineral density and that fracture risk is a concern for nonmetastatic and metastatic castration‐resistant prostate cancer.^52^

Although hormone‐related therapies for breast and prostate cancer survivors are the primary drivers of bone loss in these patients, there are other common cancer treatments that have been associated with bone loss, and the mechanism of action for bone loss sequela from these treatments has been well reviewed previously for breast and prostate cancer survivors.^18^, ^53^ Specifically, the use of traditional chemotherapies such as cisplatin, ifosfamide, cyclophosphamide, doxorubicin, and methotrexate all have bone‐related side effects. Newer targeted therapies are also emerging as having significant bone loss sequela.

## Muscle Loss in Cancer Patients

Muscle wasting is a commonly observed phenomenon in the setting of cancer.^54^ As muscle is lost, patients may initially be considered sarcopenic, a term taken from Greek *sárks penia*, a poverty of flesh. If muscle loss continues, patients may be diagnosed with cachexia, from the Greek *kákos* + *hexis*, a bad state of the body. Thresholds to define both sarcopenia and cachexia vary enough to result in a 19‐ to 26‐fold variation in prevalence.^55^ The clinical definitions of sarcopenia include the presence of low skeletal muscle mass and either 1) low muscle strength, 2) low muscle function, or 3) low muscle performance. For research purposes, sarcopenia may be defined as skeletal muscle index measured via CT scan, as muscle area (cm^2^) standardized to height (m^2^), with specific cutpoints by sex and body mass index (BMI).^56^ Sarcopenic obesity refers to depleted muscle mass in individuals with BMI higher than 30 kg/m^2^.^57^ Sarcopenia can be found as being along the progression toward cachexia, for which there are also multiple definitions. A recent international consensus process led by experts in medical cachexia defined cachexia as weight loss of at least 5% or more in 12 months or less in the presence of underlying illness, plus three of the following criteria: decreased muscle strength, fatigue, anorexia, low fat‐free mass index, abnormal biochemistry (increased inflammatory markers [C‐reactive protein >5.0 mg/L, IL‐6 >4.0 pg/mL], anemia [<12 g/dL], and low serum albumin [<3.5 g/dL]).^58^

Within the setting of cancer, prevalence of sarcopenia has been reported to be 16% among long‐term breast cancer survivors^59^ and 56% and 60% among women and men, respectively, with stage 1 to 3 colorectal cancer.^60^ Further, sarcopenia is not reserved to those with a BMI <25 mg/kg^2^. Among patients with solid tumors of the respiratory or gastrointestinal systems, 15% of obese patients (BMI >30 kg/m^2^) were sarcopenic.^57^ To place the prevalence of sarcopenia during cancer into context, the prevalence in the general population (defined as: [(Appendicular Skeletal Muscle Mass)/BMI] <0.512 for women, or <0.789 for men) is 12% or 34% in women and men of all ages, and 48% or 27.5% among women and men older than 80 years.^61^ Comparison of prevalence among cancer patients and the general population suggests that sarcopenia may be more prevalent among cancer patients, though direct comparisons have yet to be made.

The prevalence of cachexia varies by stage of cancer: 0.5% in all cancer patients versus 36% to 80% among advanced cancer patients.^62^ The incidence rate of cachexia is often highly associated with tumor type presentation and as such, patients with pancreatic cancer, gastric cancer, and lung cancer predominantly are reported to have higher incidence of cachexia.^63^, ^64^, ^65^ The progression of cachexia is exacerbated in the presence of metastasis. For example, in lung cancer patients with metastasis, occurrence of cachexia is higher in metastatic patients than in nonmetastatic patients.^66^ As with sarcopenia, cachexia is not limited to those with a BMI <25. In one study, 35.9% of advanced cancer patients were diagnosed as cachectic. Among those, 58% and 14% were normal weight and overweight/obese, respectively.^67^

It is difficult to assess the impact of chemotherapy directly on skeletal muscle in patients, but one study suggests that patients treated with neoadjuvant chemotherapy experience significant muscle wasting.^68^ In addition to loss of muscle mass, muscle weakness is an equally important adverse effect of cancer and cancer therapy (chemotherapy, radiation, and hormone‐deprivation) in cancer patients. This aspect is gaining attention as clinicians are beginning to directly assess physical activity and functional status in their patients. For example, breast cancer patients report impaired muscle function when compared with healthy peers.^69^ A recent assessment of the functional capacity of breast cancer patients (stationary bicycle measure of power) showed that loss of muscle function was independent of loss of muscle mass.^70^ The clinical impact of muscle weakness in cancer needs to be more thoroughly investigated because outright cachexia represents one end of a very extreme spectrum with muscle weakness but not wasting on one end and muscle weakness with severe cachexia on the other end.

Many animal studies have shown that chemotherapy causes skeletal muscle atrophy. The common chemotherapy, cisplatin, has been shown to cause muscle atrophy that is associated with activation of NF‐κB signaling pathway and independent of the well‐characterized activation of ubiquitin proteosomal degradation.^71^ Another platinum coordinating therapy, carboplatin, has also been shown to lead to muscle wasting.^20^ Doxorubicin causes skeletal muscle weakness in part through a tumor necrosis factor receptor (TNFR1)‐dependent manner.^72^, ^73^ Finally, the combination therapy, Folfiri, has been shown to lead to skeletal muscle wasting that includes mitochondrial dysfunction.^74^

Multiple studies have examined the impact of muscle loss on cancer‐specific survival (CSS), disease‐free survival (DFS), and overall survival (OS), as well as time to tumor progression and chemotoxicities. For example, Prado and colleagues noted that sarcopenic women with metastatic breast cancer who were treated with capecitabine had a shorter time to tumor progression as well as elevated treatment toxicities compared with nonsarcopenic patients.^75^ Deluche noted that early stage breast cancer patients with sarcopenia had reduced DFS and OS.^76^ Two studies have assessed the impact of sarcopenia on outcomes in hepatocellular carcinoma and observed elevations in chemotoxicities^77^ and decreased overall survival.^78^ Similar findings have been noted in gastric cancer.^79^ In a recent systematic review that included 7843 patients from 38 studies, sarcopenia was found to be predictive of worse CCS, DFS, and OS.^80^ In some clinical studies, the definition of sarcopenia and cachexia have led to significant confusion, but given these highly consistent findings that muscle loss is associated with worse cancer prognosis, explorations of possible mechanisms through which muscle loss occurs is worthy of attention.

One mechanism associated with cancer cachexia is elevated inflammation, including high levels of interleukin (IL)‐6 as well as increased oxidative stress.^81^ These same factors are also associated with the development and progression of tumors. In turn, skeletal muscle ryanodine receptor 1 (RyR1) intracellular Ca^2+^ release channels, required for skeletal muscle excitation–contraction coupling, becomes oxidized in the setting of bone metastases, resulting in reduced muscle function.^82^ Skeletal muscle samples from human lung and breast cancer patients with bone metastases also exhibit evidence of RyR1 Ca^2+^ channel leak. Remaining questions in this area include whether improvements in muscle mass, function, and strength could prevent onset or progression of cachexia.

In humans, resistance exercise has been shown to prevent increases in IL‐6 during treatment for breast cancer.^83^ Further, low‐intensity, low‐volume resistance exercise in rodents reduced inflammatory cytokines.^84^ In addition, protein supplementation boosts the increases in muscle mass observed with resistance exercise training.^85^ An ongoing randomized controlled trial in early stage human colon cancer patients is examining the potential for resistance exercise and protein supplementation during chemotherapy to increase muscle mass, decrease chronic inflammation, and improve cancer outcomes (NCT03291951).

## Exercise and Nutritional Interventions

Exercise is recommended for maintenance of bone and muscle in patients undergoing treatment for cancer.^86^, ^87^ Moderate‐intensity weight‐bearing aerobic and resistance exercise is recommended to preserve and improve bone density in adult populations with and without cancer. The American College of Sports Medicine (ACSM) specifically recommends that adults perform 30 to 60 minutes of endurance activities 3 to 5 times per week and of resistance exercise 2 to 3 times per week.^87^ Resistance and aerobic exercise training have been shown to preserve or improve bone density in cancer survivors and patients actively undergoing hormone therapy.^88^, ^89^, ^90^, ^91^, ^92^ Twenty‐six weeks of combined aerobic and resistance training has shown benefit for female cancer survivors in improved spine, hip, and whole body bone mineral density (BMD), but these results were not stratified by type of cancer or cancer therapy during primary tumor treatment.^93^ In another study, again not stratified by tumor type or treatment, 24 months of strength and weight training showed improved balance and muscle strength in breast cancer survivors.^94^ In metastatic breast cancer patients (65% of whom had bone metastases), reduced muscle strength and lower physical activity was reported compared with healthy age‐matched women.^95^ There are limited studies during chemotherapy to provide conclusive evidence on the effectiveness of exercise during chemotherapy to preserve bone health. Only two studies have been identified that assessed the effect of exercise on bone health during chemotherapy for women diagnosed with breast cancer, with both studies finding no significant effect.^16^, ^96^ However, neither study met the ACSM guidelines for exercise to maintain bone health.

Progressive resistance training has been shown to increase lean muscle mass and muscular strength in patients diagnosed with cancer, both during and after treatment.^97^, ^98^, ^99^ As such, exercise has been suggested as a therapeutic strategy to prevent or treat cancer‐related cachexia; however, there is limited clinical evidence supporting this suggestion.^100^ A confounding factor to most of the reported studies is that the exercise regimen (aerobic or resistance) is not consistent, nor is the duration or intensity. To get a better handle on the true effects, better controlled studies are needed in the appropriate patient populations (eg, precachexia) to determine the effect and dosage of exercise on bone health during chemotherapy treatment as well as in the prevention and treatment of cancer‐related cachexia.

Nutrition is important in the preservation of bone and muscle mass for patients diagnosed with cancer. Calcium and vitamin D supplements are potential strategies to prevent bone loss in individuals undergoing treatment for cancer. Individuals at risk for bone loss are recommended to consume 1200 to 1500 mg of calcium and 400 to 600 IU of vitamin D per day; however, there are no established guidelines for calcium and vitamin D in patients with cancer.^86^, ^101^ Studies have shown that 70% to 97.5% of patients with cancer are vitamin D deficient.^102^, ^103^, ^104^, ^105^ Calcium levels in patients with cancer vary. Up to 3% of patients with cancer experience hypercalcemia, with the majority of these patients having an advanced cancer diagnosis.^106^, ^107^ The increased calcium levels are typically due to increased parathyroid hormone–related protein (PTHrP) levels secreted by the tumor.^108^ PTHrP increases the bone‐resorbing activity of osteoclasts resulting in the hypercalcemia found in these patients. In contrast, hypocalcemia occurs as a result of osteoblastic lesions or the use of bone‐modifying agents such as denosumab or bisphosphonate. Few studies have looked at the total incidence of hypocalcemia in this patient population; however, a recent systematic review found that 5% of patients with cancer treated with denosumab develop hypocalcemia.^109^ No data have been published on the incidence and severity of either hyper‐ or hypocalcemia in patients with early stage cancer who are not receiving bone‐modifying agents. Two systematic reviews looking at the effect of calcium and vitamin D supplementation on bone health in early stage breast and prostate cancer patients receiving hormone therapy found the current recommended guidelines to be inadequate to have an effect on bone health.^110^, ^111^ Further studies are required to establish the efficacy of and guidelines for calcium and vitamin D consumption or supplementation on bone health in this population.

Cancer‐related cachexia is not reversible by conventional oral nutritional support.^112^ Parenteral nutrition and supplementation with branched chain amino acids and fish oil have shown promising results in preserving muscle mass loss in patients at risk for developing cachexia.^113^, ^114^, ^115^, ^116^ Interventions to increase amino acid ingestions to promote protein synthesis in patients diagnosed with cachexia have shown conflicting results.^117^, ^118^ Further clinical studies are needed to fully elucidate the effects of these interventions on muscle mass and survival in patients with cancer‐related cachexia and make meaningful progress for patients.

## Preclinical Models of Advanced Metastatic Cancer and Cachexia

Advanced cancers with metastases are the most dangerous to patients and many animal models have been developed to study tumor metastasis. Our focus in this review is on metastatic spread that includes bone. Bone metastases are common in advanced cancer such as breast, prostate, and lung cancer and associated with bone pain, fracture, hypercalcemia, and muscle weakness.^119^, ^120^ Bone‐muscle cross‐talk is a key nexus in the development of muscle weakness in bone metastases^121^ and may be equally important in the development and progression of cachexia. The endocrine signals between bone and muscle are of great interest and likely play a large role in the overall health of the musculoskeletal system. Bone is a uniquely capable tissue for nascent tumor cell survival and proliferation. Solid tumor metastases to bone stimulate bone destruction via osteoclast‐mediated bone resorption, releasing active transforming growth factor β (TGFβ) stored in the bone matrix to promote a feed‐forward cycle of tumor growth and bone destruction.^120^, ^122^, ^123^, ^124^, ^125^, ^126^, ^127^

We were the first to describe cancer cachexia in animal models of osteolytic bone metastases (human MDA‐MB‐231 cells metastatic to bone). We identified mechanism of bone‐muscle cross‐talk by which bone‐derived TGFβ contributes to muscle weakness via oxidation of skeletal muscle ryanodine receptor1 Ca^2+^ release channel that is critical for excitation‐contraction coupling (E‐C coupling).^121^ It has been previously shown that leaky skeletal muscle RyR1 Ca^2+^ channels cause muscle weakness.^128^, ^129^, ^130^ This RyR1 channel Ca^2+^ leak results in a reduction in the amount of Ca^2+^ stored in the sarcoplasmic reticulum, which directly reduces the force of skeletal muscle contraction because it is dependent on the level of tetanic Ca^2+^ released from the sarcoplasmic reticulum. In support of the bone microenvironment's systemic effects that promote muscle weakness in osteolytic cancer in bone, and that TGFβ is a major mediator, we have shown 1) increased serum TGFβ concentrations in mice with breast cancer bone metastases compared with mice with primary breast cancer (no bone metastases); 2) TGFβ activity increased in skeletal muscle from mice and humans with bone metastases; 3) TGFβ activity is reduced by antiresorptive therapy (bisphosphonate); 4) skeletal muscle expression of NADPH oxidase 4 (Nox4) increased and Nox4‐RyR1 binding increased in muscle from mice and humans with bone metastases; and 5) increased oxidation of skeletal muscle RyR1 and RyR1 Ca^2+^ leak. Nox4 is a constitutively active oxidase and TGFβ target gene that generates reactive oxygen species (ROS).^131^, ^132^ Oxidation of RyR1 results in sarcoplasmic reticulum Ca^2+^ leak and reduced muscle strength due to loss of the RyR1 complex stabilizing subunit, calstabin1. Calstabin1 maintains the closed state of the RyR1 channel. RyR1 oxidation and decreased calstabin1 binding is a biochemical signature associated with RyR1 sarcoplasmic reticulum Ca^2+^ leak.^128^, ^129^ These are direct effects of osteolytic bone metastases and not due to presence of tumor cells. In fact, a 10‐fold larger inoculum of MDA‐MB‐231 cells into the primary site (mammary fat pad) did not induce cachexia, skeletal muscle weakness, TGFβ signaling in muscle, increased oxidative stress, or RyR1 Ca^2+^ leak.^121^

A number of other murine metastatic models have been used to evaluate the biologic changes in bone induced by tumor cells. These include xenografts, either primary cells from patients or established human cell lines, and transplanted into immunocompromised murine hosts.^133^ Xenograph models have been useful to elucidate mechanistic events within the tumor cells and within osteocytes, and as described, above cancer cachexia in osteolytic bone metastases. However, the absence of relevant stromal‐tumor interactions and a functional immune system can be limitations to these models, depending on experimental questions. Syngeneic mouse models of metastatic breast cancer have been used with some success to evaluate stromal‐tumor interactions and the role of the immune system in controlling metastatic progression but to a lesser extent cancer cachexia. In particular, in transplantable models, tumor cells can be injected orthotopically into the mammary gland into a syngeneic host, and primary tumor growth and metastatic progression occur within weeks to months.^134^ These models have been established from tumor cells isolated from spontaneous mammary tumors in mice.^135^, ^136^ The 4T1 model, a collection of mammary tumor cell lines syngeneic in BALB/c mice, has been the principal transplantable mouse model used to study both tumor‐ and host‐derived factors involved in spontaneous metastasis.^135^, ^137^, ^138^ However, recently the E0771 metastatic model has been developed in C57/BL6 mice.^136^ Each of these models has specific strengths depending on the scientific question but as a group represent a power set of tools to unravel bone and muscle pathology in cancer and cancer therapy.

## Potential Pharmacological and Nonpharmacological Therapeutics

As described above, mice with breast cancer bone metastases develop significant skeletal muscle weakness and cachexia. Stabilization of RyR1 with the small molecule, Rycal S107, prevents RyR1 Ca^2+^ leak even in the presence of oxidative stress by preventing loss of calstabin1 and did not affect tumor burden. Further, either inhibiting TGFβ directly (TGFβ receptor blockade [SD208] or TGFβ neutralizing antibody [1D11]) or indirectly (blocking TGFβ release from bone using a bisphosphonate zoledronic acid [ZA]) prevented RyR1 oxidation and restored muscle strength in mice with breast cancer bone metastases. Blockade of Nox4 (GKT137831) also restored skeletal muscle strength without affecting tumor burden. Finally, human muscle samples taken from patients with breast cancer and lung cancer with bone metastases had oxidation of RyR1 and loss of calstabin1, validating the clinical significance of these data.^121^ Thus, TGFβ released from the tumor‐bone microenvironment promotes oxidation of RyR1 and contributes to cancer‐associated skeletal muscle weakness.

The potential of these targets for translation in the clinic is high. Bisphosphonates, such as ZA, are already FDA‐approved. GKT137831 has recently completed phase 2 clinical trials for diabetic nephropathy (trial no. NCT02010242). Anti‐TGFβ therapies have been widely tested across multiple diseases, including recently for metastatic breast cancer in a phase 2 trial by Eli Lilly (trial no. NCT02538471). Finally, a clinical version of Rycal S107 (ARM210/S48168) has obtained FDA Orphan Drug Designation and Rare Pediatric Disease Designation for the treatment of Duchenne muscular dystrophy. Combining these novel therapeutics could lead to improvement for patients with cancer‐associated skeletal muscle weakness.

Other important regulators of cachexia (eg, TNFα, IL‐6) could lead to muscle weakness in osteolytic cancer in bone. Indeed, reduced contractile protein expression or function may also contribute to weakness in bone metastases and could be involved in the novel link we described between bone destruction and weakness via the TGFβ‐Nox4‐RyR1 axis.^121^ Mice with breast cancer bone metastases also exhibit severe cachexia in addition to TGFβ‐mediated Ca^2+^ mishandling, after initial muscle weakness is detected.^121^ Other factors released from the bone matrix during osteolytic bone destruction, such as activin, may contribute to muscle atrophy that is independent of RyR1 Ca^2+^ leak and remains to be investigated.

In addition to pharmacologic targets, exercise may yield significant gains in musculoskeletal function. The mechanism(s) of exercise‐induced modulation of bone‐muscle cross‐talk in the setting of metastatic cancer patients is an evolving field. In preclinical models, we have demonstrated that 8 weeks of voluntary wheel running, a form of aerobic exercise in mice, can significantly reduce metastatic burden in the lungs and femurs using a clone in the 4T1 series (4T1.2 transfected with luciferase) that has a predilection for bone (unpublished results). The mechanism underlying the protective effect of exercise on metastases is not completely understood. Exercise may be preventing dissemination of tumor cells from the primary tumor or altering the trafficking of metastases into tissue. The entry of tumor cells into the bone microenvironment may cause a number of significant changes including an increase in growth factors (eg, parathyroid hormone–related protein), which causes an upregulation of RANKL, and downregulation of osteoprotegerin (OPG),^123^ which enhances osteoclast function resulting in greater bone degradation. As a consequence, TGF‐β, VEGF, IGF‐1, and other bone morphogenetic proteins are released into the bone microenvironment.^139^ In addition, tumor cells secrete cytokine such as IL‐6, IL‐8, and MCP‐1 that impact osteoclast differentiation.^140^ Exercise may be interrupting a number of the aforementioned pathways to reduce the metastatic burden in bone. In addition, aerobic and/or resistance exercise may be useful interventions to prevent or delay cancer cachexia in osteolytic bone metastases because of the reduction in inflammatory cytokines or improvement in metabolic outcomes observed with exercise. The evaluation of both aerobic and resistance exercise in the 4T1 model may allow us to evaluate exercise as a therapeutic intervention to prevent cancer cachexia and the key biological mediators involved.

Clinical trials have shown that exercise improves bone density in patients with metastatic bone lesions.^141^, ^142^ Improvements in bone density in this patient population have been associated with a significant decrease in serum markers of bone resorption such as pyridinium cross‐links pyridinoline (PYD) and C‐terminal cross‐linking telopeptide of type I collagen (CTX‐1).^142^ Although mechanical loading of bone and/or dietary components have well‐established mechanistic effects on osteoclast and osteoblast activity,^143^ it is unknown if such mechanisms translate to cachectic conditions often found in patients with bone lesions. Further, the utility of exercise as a nonpharmacological intervention for muscle mass and muscle function appears to be most beneficial in patients with metastatic cancer who are precachectic. Exercise has been shown to decrease circulating levels of IL‐6 in patients with a cancer diagnosis.^83^ Additionally, resistance training upregulates IGF‐1, which results in activation of the downstream pathway IRS1/PI3K/Akt, and aerobic exercise has been shown to increase production of PGC‐1α4.^144^ Both Akt and PGC‐1α4 inhibit FOXO3, an upregulator of two ubiquitin ligases important in sarcomere breakdown and inhibition of protein synthesis.^81^, ^145^ An important caveat to the above exercise clinical trials in patients with metastatic bone lesions is to recognize that patients were healthy enough to exercise and had limited pain and or sufficient pain management, thus impacting their choice to volunteer for an exercise study.^83^, ^141^, ^142^, ^145^ Although exercise can be tailored in many different ways, a patient's medical history, clinical presentation (comorbidities and pain), as well as previous health behaviors need to be taken into consideration for treatment choice.

In addition to the effects of exercise on musculoskeletal outcomes, exercise after a cancer diagnosis reduces the risk of cancer recurrence and improves both cancer‐specific and all‐cause mortality. Numerous meta‐analyses^146^, ^147^, ^148^, ^149^, ^150^, ^151^, ^152^, ^153^, ^154^, ^155^ and a recent systematic review^156^ have evaluated the strength of the evidence, and there are a considerable number of studies demonstrating a protective effect of exercise on recurrence and mortality in breast, colorectal, and prostate cancer patients. Specifically, exercise after a cancer diagnosis was associated with a 21% to 35% lower risk of cancer recurrence, a 28% to 44% lower risk of cancer‐specific mortality, and a 25% to 48% decreased risk of all‐cause mortality.^156^ In the aforementioned studies, exercise patterns were assessed by using a variety of self‐report and interview‐administered questionnaires that evaluated a range of exercise habits (eg, occupational, recreational). To date, it is unclear whether exercise is associated with improvements in recurrence and/or survival in patients diagnosed with other cancer types. Furthermore, more studies are need to determine the type of exercise and the dose, duration, and frequency of exercise that is the most beneficial for improving cancer outcomes. Exercise also improves quality of life in some but not all cancer survivors. Improvements in quality of life with exercise have been observed in breast cancer and hematological malignancies, but not in prostate, lung, colorectal, or gynecological cancer survivors.^(156)^ The lack of a positive effect of exercise on quality of life in some cancer may be driven by a limited number of studies in these types of survivors and warrants further study.

## Summary

**Figure 1:**
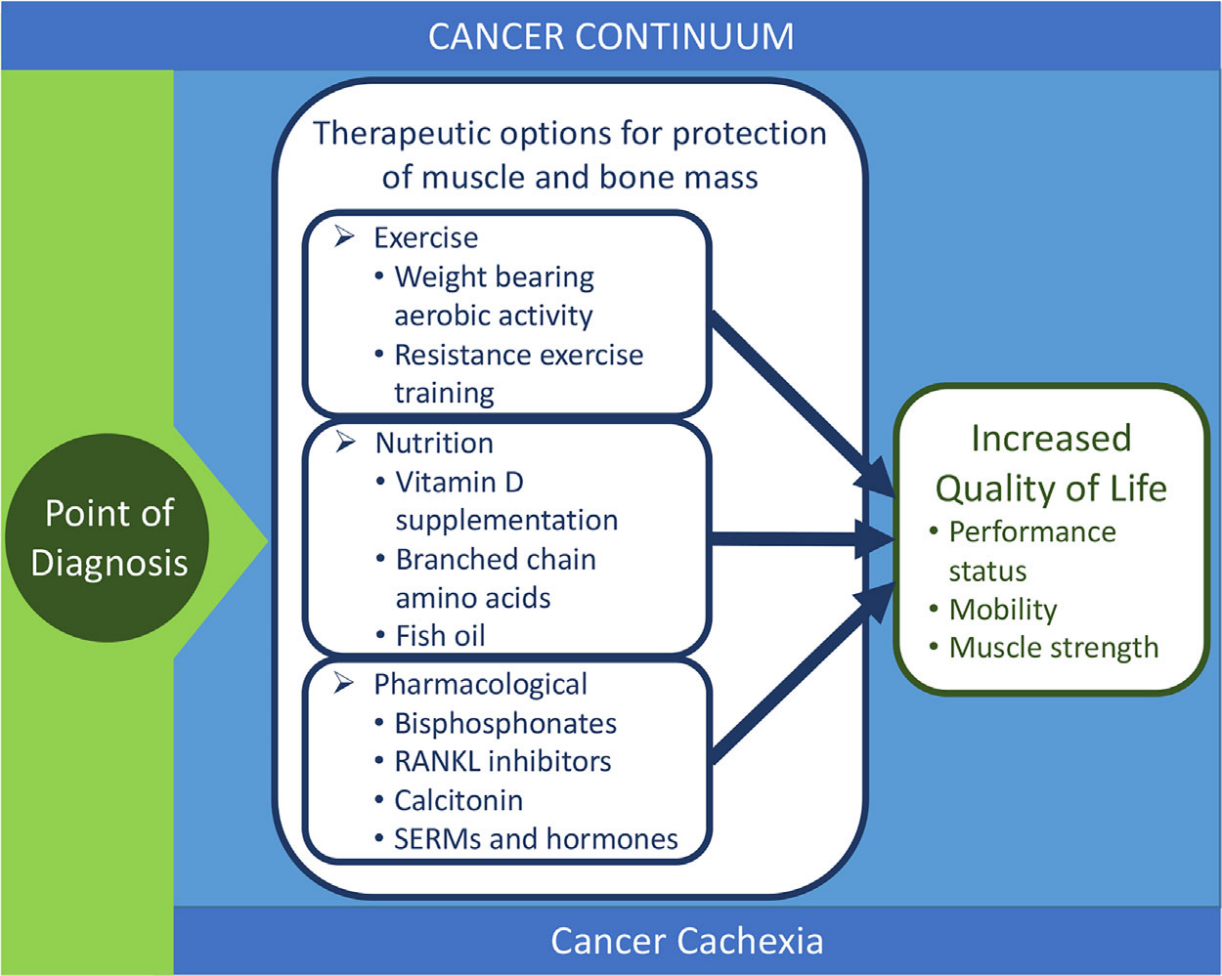
Schematic showing the therapeutic options for loss of bone and muscle throughout the cancer continuum.

Erosion of musculoskeletal function severely impacts quality of life. In cancer patients, the loss of mobility and risk for falls and fractures are a major concern and can be caused by the tumor itself but also by the therapies used to reduce tumor burden. The loss of muscle mass and function (cachexia) and the loss of bone mass are connected in a feedback loop due to the tight interconnected mechanical and endocrine functions of these tissues. The cancer continuum, from the point of diagnosis to treatment, and survivorship is a highly variable process. For certain cancers, therapeutic strategies do try to account for musculoskeletal effects. In pancreatic cancer, for example, where cachexia incidence is high, nutrition is a key focus during treatment. In bone metastases, the use of bisphosphonates or denosumab to protect against bone loss is used in conjunction with antitumor therapies. However, most treatment strategies do not fully incorporate protection of the musculoskeletal system even though this is critical to quality of life and survival (Fig. 1). A key future direction is the incorporation of musculoskeletal protection and improved function early in the cancer continuum.

## Disclosures

All authors state that they do not have any conflicts of interest.
